# Misconceptions on the use of MR-Egger regression and the evaluation of the InSIDE assumption

**DOI:** 10.1093/ije/dyx192

**Published:** 2017-09-12

**Authors:** Jack Bowden

**Affiliations:** MRC Integrative Epidemiology Unit at the University of Bristol, Oakfield House, Oakfield Grove, Bristol BS8 2BN, UK.

In their letter to this journal, Slob *et al.*^1^ attempt to derive the bias of the MR-Egger regression^2^ estimate for a Mendelian randomization (MR) analysis. They show that its bias can be larger than that of the inverse variance weighted (IVW) estimate when the instrument strength independent of direct effect (InSIDE) assumption is violated, and suggest a method for assessing the magnitude of InSIDE violation in any given data set. Slob *et al.* conclude by cautioning against placing undue reliance on the MR-Egger estimate in practice.

Whereas I agree with the basic sentiment of their letter, I wish to make several minor points of correction and clarification. I must also highlight a major flaw in their argument concerning a test for InSIDE violation, so that it is not subsequently repeated by others.

I would not recommend the use of MR-Egger regression, in its current form, in the `single sample’ setting, that is when genetic associations with the exposure and with the outcome are measured in the same subjects. This viewpoint is put forward in my reply^3^ to a recent letter by Hartwig and Davies^4^ to the *IJE.*

Slob *et al.*^1^ helpfully state that the asymptotic bias of the inverse variance weighted (IVW) and MR-Egger estimates (or equivalently their underlying estimands) has in fact already been derived by Bowden *et al.*,^5^ specifically in equations (23) and (24). Unfortunately, the expressions given in Slob *et al.*^1^ and referenced to Bowden *et al.*^5^ do not match, and I have some concerns as to their validity. For example, the expression given by Slob *et al.* for the bias of the IVW estimate depends on the parameter estimate for the instrument-exposure association. This is at odds with the very definition of bias as an expected value of a random variable minus its target parameter. It is hard to ascertain whether the expression for the bias of the MR-Egger estimate is correct, as no derivation is given. However, the denominator of their expression ($\sigma_{\gamma }$) is confusing because it should surely be a function of the direct effect of the IV on the exposure, represented by γ, and the indirect effect of the IV on the exposure, represented by φ.

In Bowden *et al.*,^5^ we show that the MR-Egger estimate can indeed be more biased than the IVW estimate when InSIDE is violated, especially when the mean pleiotropic effect is close to zero and there is little variation in the single nucleotide polymorphism (SNP) exposure association estimates. For this reason, alternative pleiotropy-robust estimation strategies, such as the weighted median^6^ and the mode-based estimate,^7^ have been proposed that do not rely on the InSIDE assumption and therefore naturally complement MR-Egger in a sensitivity analysis.

Several statistics have also been proposed to evaluate the suitability of MR-Egger regression in two-sample MR studies. The first is the $I_{GX}^{2}$ statistic,^8^ which quantifies the notion of instrument strength for MR-Egger, and gives an indication of its `weak instrument’ bias. We recommend that $I_{GX}^{2}$ should be high (e.g. as close to 1 as possible) for the set of variants in an MR study, in order to be capable of furnishing a reliable MR-Egger causal estimate. Briefly this requires that the SNP exposure association estimates are both precise but sufficiently varied. If it had been correctly stated, it would make the denominator of *Slob et al.*’s bias expression for MR-Egger large and hence the bias small.

A second statistic,$Q_{R}$, introduced in Bowden *et al.*,^5^ quantifies the relative goodness of fit of MR-Egger over the IVW approach. Specifically, it is the ratio of the statistical heterogeneity around the MR-Egger fitted slope, divided by the statistical heterogeneity around the IVW slope. A $Q_{R}$ close to 1 indicates that MR-Egger is not a better fit to the data and therefore offers no benefit over IVW whatsoever, given its relative lack of precision. Conversely, a $Q_{R}$much less than 1 indicates that MR-Egger is a better fit to the data and its estimate should be taken seriously. We recommend careful and considered use of $I_{GX}^{2}$and $Q_{R}$ to help identify cases where MR-Egger should be used, or indeed avoided.

Slob *et al.* propose to estimate the degree of violation of the InSIDE assumption, by first using the IVW estimate as a proxy for the true causal estimate to calculate individual pleiotropic effects for each variant. I fundamentally disagree with this analysis because it employs circular reasoning: the IVW estimate is generally biased for the causal effect, precisely because of pleiotropy, whenever it has a non-zero mean. To see this, assume for simplicity the following linear model linking L single nucleotide polymorphism (SNP) outcome association parameters, Γ, to their corresponding SNP exposure association parameters, γ, and pleiotropic effect parameters, α:

(1) $$\Gamma_{j}=\alpha_{j}+\beta \gamma_{j},\;j=1,...,L$$

Here β is the causal effect parameter. Model (1) allows us to see what quantities different estimators (e.g. IVW, MR-Egger) target asymptotically (i.e. their estimands) as the sample size grows large. We will assume that the genetic data have been coded so that the SNP exposure association parameters are positive. Assume also for simplicity, but without loss of generality, that the IVW estimand is a weighted average of ratio estimands $\beta_{j}=$ $\frac{\Gamma_{j}}{\gamma_{j}}$, where the weights are equal to $\gamma_{j}^{2}$ (as would be the case if the SNPs had identical allele frequency), that is:

(2) $$\beta_{IVW}=\frac{\sum_{j=1}^{L}\beta_{j}\gamma_{j}^{2}}{\sum_{j=1}^{L}\gamma_{j}^{2}}=\beta +\frac{\sum_{j=1}^{L}\alpha_{j}\gamma_{j}}{\sum_{j=1}^{L}\gamma_{j}^{2}}$$

The second term on the right hand side of equation (2) represents the asymptotic bias of the IVW estimate. Consider the numerator of this bias term. It is zero whenever the sample covariance of $\alpha_{j}$ and $\gamma_{j}$, $S_{\alpha ,\gamma }$ say, and the product of their means, $\overset{¯}{\alpha }.\overset{¯}{\gamma }$ say, is zero. That is, if:

(3) $$S_{\alpha ,\gamma }+\overset{¯}{\alpha }.\overset{¯}{\gamma }=0$$

Therefore, formula (3) makes clear that $\beta_{IVW}$ is only equal to β in general when (i) the InSIDE assumption holds perfectly (so $S_{\alpha ,\gamma }$ is zero) and (ii) the mean pleiotropic effect $\overset{¯}{\alpha }$ is zero (we have already ruled out the possibility that $\overset{¯}{\gamma }$ is zero). Of course, both (i) and (ii) may be false and equation (3) still equal zero in the case where one perfectly cancels out the other.

When Slob *et al.* attempt to estimate the pleiotropy parameters by plugging the IVW estimate given in formula (2) into equation (1), and then look to see if they are correlated with the SNP exposure associations, they are instead evaluating the correlation between $\gamma_{j}$ and

(4) $$\alpha_{j}-\gamma_{j}\frac{\sum_{j=1}^{L}\alpha_{j}\gamma_{j}}{\sum_{j=1}^{L}\gamma_{j}^{2}}.$$

However, these quantities are clearly correlated whenever equation (3) is non-zero. For example, when the InSIDE assumption is satisfied but $\overset{¯}{\alpha }$ happens to be non-zero. The correlations calculated by Slob *et al.* in their two examples were both negative. Formula (3) and formula (4) imply that the mean pleiotropic effect $\overset{¯}{\alpha }$ must have been positive in each case.

In contrast to the IVW estimate, MR-Egger regression only relies on the InSIDE assumption and not additionally on non-zero mean pleiotropy. Indeed, it exploits InSIDE to identify, estimate and adjust for non-zero mean pleiotropy.

Slob *et al.* note that the correlation between their estimated pleiotropic effects and instrument strength is reduced when using the MR-Egger estimate as opposed to the IVW estimate in place of the causal effect. It is easy to show that it should be identical to zero. That it is not zero in their examples is probably a reflection of the fact that they have estimated the MR-Egger regression coefficients via a weighted analysis (e.g. by accounting for differing allele frequencies), but evaluated the correlation in an unweighted fashion.

The letter by Slob *et al.*^1^ re-states some facts already in the public domain,^5^ but unfortunately it contains several minor inaccuracies and one serious, unhelpful misconception. I would strongly discourage researchers from using the IVW estimate to quantify the magnitude of InSIDE violation and to assess the relative bias of the IVW and MR-Egger estimates, because the IVW estimate also requires the InSIDE assumption. This is explained in Bowden *et al.*^5^

If a reliable test for violation of the InSIDE assumption could be developed, it would be extremely useful for determining the reliability of the IVW and MR-Egger estimates, and would be of great importance to the field of Mendelian randomization. Unfortunately, the method proposed by Slob *et al.* is flawed. Other authors have also recently developed informal strategies for testing InSIDE^9^ that have been shown to be unreliable.^10^

In my opinion, external data of some sort are required to test the InSIDE assumption. Multivariable Mendelian randomization methods,^11^ and future extensions thereof, are a promising avenue of research in this regard.
